# An investigation of the intention and reasons of senior high school students in China to choose medical school

**DOI:** 10.1186/s12909-021-02677-w

**Published:** 2021-04-26

**Authors:** Chaoqun Yang, Xuerui Jin, Ji Yan, Junkai Zhang, Canyu Chen, Yaqing Cheng, Jialin You, Guoying Deng

**Affiliations:** 1 Trauma Center, Shanghai General Hospital, Shanghai Jiaotong University School of Medicine, No. 650 Xin Songjiang Road, Shanghai, China; 2 Shanghai Jiao Tong University School of Medicine, Shanghai, 200025 China; 3 Youth League Committee Office, Shanghai Jiao Tong University School of Medicine, Shanghai, 200025 China

## Abstract

**Background:**

Shortages of qualified health workers have been a global concern, especially in developing countries. China also faces this dilemma, which hinders the development of public health services. Senior high school students are a group who are considering their college majors and careers after graduation. They are also a potential and basic talent reserve for the health sector. This survey focused on senior high school students’ intention to learn clinical medicine and explored potential influencing factors.

**Methods:**

An anonymous questionnaire containing 20 items was distributed to 5344 senior high school students. The questions covered the following topics: students’ intention to learn clinical medicine, personal and family information, understanding of medical education, cognition of doctors’ working conditions, and doctor-patient relationships. Logistic regression and the chi-square test were used to compare students with and without a clear intention to learn clinical medicine to explore influencing factors.

**Results:**

Only 5.6% of senior high school students had a clear intention to learn medicine (CILCM). Personal and family information had distinct impacts. Interest and anatomy course were also associated with students’ choice. There was a positive correlation between understanding of medical education and students’ intention Meanwhile, students’ cognition of doctors, career prospects, and social status had significant impacts. The more optimistic students were about doctors’ working conditions and doctor-patient relationships, the more likely they were to have a CILCM.

**Conclusion:**

To some extent, this survey reflects the shortage of medical talent in China and provides possible clues for solving this problem. In addition, these findings may provide a perspective for understanding the development of health services in developing countries.

**Supplementary Information:**

The online version contains supplementary material available at 10.1186/s12909-021-02677-w.

## Background

The World Health Organization (WHO) estimated a global deficit of nearly 4.3 million health workers: a critical shortage is faced by 57 countries [1]. A model predicts that global demand for health workers will rise to 80 million workers, resulting in a worldwide net shortage of 15 million health workers by 2030 [2]. The shortage of qualified health workers has been a global concern, especially in developing countries. Due to relatively robust economic growth, rapid population growth, and ageing, shortages are predicted to be the most severe in middle-income countries and the East Asia and Pacific region [2, 3]. For example, in China, the health workforce has 1.9 doctors per 1000 individuals, which is lower than the UK’s rate of 2.8 and the USA’s of 2.4 per 1000 [4]. Among the global population in 2017, nearly a quarter of the population over 60 was living in China; the population over 60 in China is estimated to increase to 478.9 million people by 2050 [5]. The significant increase in the ageing population will lead to a substantial increase in demand for health workers [6].

Over the last decades, Chinese government have launched a comprehensive healthcare reform to tackle challenges of a shortage of health workers and have been advancement in different fields [7]. With the requirement of Universal health coverage(UHC) and technological advancement, it is gravely important to improve the quantities and qualities of health workers, mostly doctors. However, China is facing serious problems with retention of doctors. From the beginning of 2005 to the end of 2014, only 752,233 (15.91%) of Chinese clinical medical graduates registered in practice, while the proportion of young doctors declined, and that of doctors over 60 increased [8]. The shortage of doctors and doctors’ low level of educational qualifications have hindered the development of public health services. Attracting and cultivating more high-level, application-oriented public doctors is needed. Therefore, this study will focus on clinical medicine (clinical medicine graduates are qualified to become doctors).

One review published in 2018 identified that the main motivators to select medicine in medical students of upper-middle income countries are job security, social status, and parental wish [9]. Other factors influencing the choice of medical study include interest in the medical field, good job opportunities, a desire to serve others, medical background of parents, and many more [10, 11]. Most studies addressing the motivation for studying medicine have gathered information retrospectively from medical students or even physicians. Nevertheless, senior high school students, the source of future health workers, have not been well studied in terms of their medical career choices.

In China, students can enter medical school directly after finishing secondary education, which is true in countries such as Brazil, France, Georgia ect [12]. Before being admitted to medical school, Chinese students will take the national college entrance examination (CEE) [13]. Just as other majors, students are admitted to medical schools based on whether they selected a related major and their CEE scores [14]. Students of clinical medicine enroll directly from high school for degrees that require 5 years (bachelor) or 8 years (MD) [15]. Whether senior high school students apply for clinical medicine and their characteristics directly affect the quantity and quality of future medical students and doctors.

Therefore, in this study, we systematically explored the current situation of senior high school students’ intention to learn clinical medicine and identified characteristics of those with a clear intention to learn clinical medicine (CILCM) and its associated factors. This study aimed to provide a comprehensive insight into the medical career choice of high school students and an evidence base for medical educators to intervene in the phenomenon. The findings of this study are also beneficial to guide medical colleges to attract more quality senior high school students. Furthermore, these findings provide a perspective for understanding the development status of health services in developing countries to a certain extent. Based on the aforementioned discussion, we considered students’ demographics, family background, understanding of current medical education, cognition of healthcare occupation, and doctor-patient relationship (DPR).

## Methods

### Participants and procedures

This cross-sectional survey was conducted in Shanghai, Zhejiang, Jiangxi, and Guizhou Provinces in China from March to April 2019. Respondents from 10 senior high schools in these four provinces were selected by cluster sampling. Hard copies of the questionnaire were distributed to students and collected anonymously. A total of 5344 senior high school students were targeted. The study was approved by the Ethics Committee of Shanghai General Hospital. Participation in the research was voluntary, before completing the questionnaire, the respondents confirmed that they thoroughly understood the precautions.

### Questionnaire

We developed the questionnaire based partly on existing questionnaires [16–18]. The questionnaire were screened for content and construct validity by experts. And additional items were added on the basis of interviewing with experts. For further optimize the questionnaire and response process validation, 250 senior high school students in Shanghai were invited for a pilot study. Students were asked to fill out the questionnaire. They could get answers to any questions related to the questionnaire from researchers. Immediately upon completion of the survey, some students were interviewed by the investigators using pre-determined verbal probes. Data results and students’ feedback of pilot study was an important basis of questionnaire optimization and revision. The final version of the questionnaire consisted of 20 closed-ended questions arbitrarily divided into three parts. The first part of the questionnaire related to students’ socio-demographic details and family background including gender, grade, academic performance, whether they belonged to a medical family, whether they had parental support, their parents’ occupation, and their parents’ educational background. The second part was aimed at students’ understanding of the duration of clinical medicine schooling, interest in medicine, and whether special medical courses (anatomy, animal experiments, and so on) affect their choices. The third part examined students’ cognition of the healthcare occupation and DPR. The questionnaire consisted of 12 fixed-response questions and 8 five-point Likert scale items. In the pre-test, the questionnaire showed good test-retest reliability of 0.912, and the retest interval was two weeks. Data from the completed survey was extracted and anonymized for analysis.

### Statistical analysis

The data were analysed using Statistical Package for Social Sciences software (Version 21.0, SPSS Inc., Chicago, IL, USA). According to the respondents’ opinions on “will you apply for clinical medicine”, students who chose “definitely I will” were defined as having a clear intention to learn clinical medicine (CILCM). Respectively, students chose other options, including “maybe”, “definitely I won’t” and “I have no idea”, were defined as not having a clear intention to learn clinical medicine. Comparisons were made between students with or without a CILCM. Frequency and percentage were calculated as descriptive statistics. As categorical variables, students’ socio-demographic details and family background were analysed with the chi-square test. Bonferroni correction was applied. Univariate and multiple logistic regression analysis was used to assess students’ understanding of clinical medical education, their cognition of healthcare occupations and DPR. Statistical significance was set at *p* < 0.05 on logistic regression.

## Results

### Sample characteristics

The characteristics of the study sample are shown in Table 1. A total of 5344 students completed questionnaires with a response rate of 89.1% (5344/6000). All items were fully completed. Among them, 2502 (46.8%) were in senior year one, 1538 (28.8%) in senior year two, and 1304 (24.4%) in senior year three. A total of 52.7% (2817/5344) of the participants were female. Among the respondents, 300 (5.6%) had a CILCM.

**Table 1:** Characteristics of the sample and basic information

Item	N = total	Number of students with a CILCM* (Percent)	χ2	P-value
**Gender**			15.298	< 0.001
Male	2527	109 (4.3)		
Female	2817	191 (6.8)		
**Grade**			28.864	< 0.001
Senior one	2502	118 (4.7)		
Senior two	1538	70 (4.6)		
Senior three	1304	112 (8.6)		
**Performance ranking**			8.389	0.039
Top 5%	412	30 (7.3)		
6% ~ 30%	1350	82 (6.1)		
31% ~ 70%	2237	132 (5.9)		
71% ~ 100%	1345	56 (4.2)		
**Income per person in family (RMB)**			1.194	0.879
< 1000	690	39 (5.7)		
≥ 1000, < 3000	1391	81 (5.8)		
≥ 3000, < 5000	1475	76 (5.2)		
≥ 5000, < 10,000	1209	73 (6.0)		
≥ 10,000	579	31 (5.4)		
**One or both parents are medical workers**			11.339	0.001
Yes	250	26 (10.4)		
No	5094	274 (5.4)		
**Education level of parents**			6.572	0.037
Senior high school or below	3666	207 (5.6)		
Undergraduate/junior college education	1504	76 (5.1)		
Postgraduate or above	174	17 (9.8)		
**Family’s attitude towards learning medicine**			105.209	< 0.001
Support	2424	222 (9.2)		
Neutral	2673	71 (2.7)		
Oppose	247	7 (2.8)		

*CILCM = clear intention to learn clinical medicine

Students who will definitely apply for clinical medicine are defined as having a CILCM

### Personal and family information

Students’ personal and family information was considered as basic information and was shown in Table 1. Personal information included gender, grade, and academic performance ranking. Compared with males (109/2527, 4.3%), more females had a CILCM (191/2817, 6.8%). A total of 8.6% (112/1304) of the students in senior three had a CILCM, while the proportion of students with a CILCM in senior year one and that in senior year two was 4.7% (118/2502) and 4.6% (70/1538) respectively. After Bonferroni correction, there was only difference between students in senior three and other two grades. Among the students ranked in the top 5%,those with a CILCM accounted for 7.3% (30/412), higher than the other group, but *p*-value showed no significant difference between students with distinct academic performance.

Family information included four items: income, parents’ occupation, parents’ education level, and families’ attitude towards learning medicine. A significant difference was observed between students with and without one or both parents working in a medical institution. A total of 10.4% (26/250) of the students whose parents were medical workers had a CILCM, which was much higher than in the other students (274/5094, 5.4%). There was an increase in the number of students with a CILCM in the group of students whose parents were postgraduate or above (17/174, 9.8%), which was significantly higher than that in the undergraduate or junior college education group (76/1504, 5.1%). Families’ attitude towards learning medicine also greatly impacted students’ attitude. In families that supported students to learn medicine, the proportion of students with a CILCM (222/2424, 9.2%) was nearly three times greater than that in students whose families hold neutral (71/2673, 2.7%) or negative (7/247, 2.8%) attitudes.

### Understanding of clinical medicine education

The students’ understanding of clinical medical education included their interest in medicine, their understanding of the duration of medical schooling, and their attitude towards anatomy. The detailed data are shown in Table 2.

**Table 2:** Students’ understanding of clinical medicine education

Item	N = total	Number of students with a CILCM* (Percent)	Univariate regression		Multivariate regression	
			P-value	OR (95% CI)	P-value	OR (95% CI)
**Interest in medicine**			< 0.001	11.803 (9.530 ~ 14.619)	< 0.001	9.454 (7.327 ~ 11.567)
Totally uninterested	373	2 (0.5)				
Uninterested	1007	3 (0.34)				
Neutral	2419	22 (0.9)				
interested	1253	101 (8.1)				
Very interested	292	172 (58.9)				
**know the durationof schooling very well**			< 0.001	2.367 (2.104 ~ 2.663)	< 0.001	1.391 (1.203 ~ 1.608)
Strongly agree	109	26 (23.9)				
Agree	821	134 (16.3)				
Neutral	1539	78 (5.1)				
Disagree	1381	38 (2.8)				
Strongly disease	1494	24 (1.6)				
**Anatomy influence choice**						
Yes	2008	51 (2.5)		1		1
No	3336	249 (7.5)	< 0.001	3.095 (2.278 ~ 4.205)	0.001	1.808 (1.268 ~ 2.578)

*CILCM = clear intention to learn clinical medicine

Students who will definitely apply for clinical medicine are defined as having a CILCM

Univariate analysis revealed that students’ interest in medicine was significantly positively correlated with students’ CILCM (*P* < 0.001, OR = 11.803). Though only 5.5% (292/5344) of the students were very interested in medicine, 58.9% (172/292) of them had a CILCM, which is far higher than the proportion of students who were uninterested (3/1007, 0.3%), totally uninterested (2/373, 0.5%) or those who had neutral interest (22/2419, 0.9%).

There was another positive correlation between the degree of understanding of the duration of medical schooling and students’ CILCM (*P* < 0.001, OR = 2.367). The association with students’ attitude towards anatomy was also significant (*P* < 0.001, OR = 3.095). A total of 62.4% of the students said that anatomy courses did not affect their choice of major. Compared with the students who were influenced by anatomy, these students had a higher proportion of students with a CILCM.

The difference—especially the odds ratio—was narrowed when basic information was added as correction factors in multivariate analysis. Multivariate analysis indicated that those who were interested in medicine (*P* < 0.001, OR = 9.545), thoroughly understood the duration of schooling (*P* < 0.001, OR = 1.391), and were not be influenced by anatomy (*P* = 0.001, OR = 1.808) tended to have a CILCM.

### Cognition of healthcare occupation and DPR

As presented in Table 3, the students’ cognition of healthcare occupations consisted of their opinion on social status of doctors, doctor’s career prospects, workload, workload compared with income, and the essence of doctors’ work. Their view on DPR in China was directly questioned.

**Table 3:** Students’ cognition of healthcare occupation and doctor-patient relationship

Item	N = total	Number of students with a CILCM* (Percent)	Univariate regression		Multivariate regression	
			P-value	OR (95%CI)	P-value	OR (95%CI)
**Doctor’sareer prospects**			< 0.001	1.780 (1.496 ~ 2.119)	< 0.001	1.664 (1.371 ~ 2.021)
Very pessimistic	28	3 (10.7)				
Pessimistic	125	6 (4.8)				
Neutral	1148	29 (9.7)				
Optimistic	2999	156 (5.2)				
Very optimistic	1044	106 (35.3)				
**Social status**			0.571	0.953 (0.806 ~ 1.126)	0.013	1.254 (1.080 ~ 1.501)
Very high	465	43 (9.2)				
High	3052	149 (4.9)				
Average	1616	91 (5.6)				
Low	168	15 (8.9)				
Very low	43	2 (4.7)				
**Workload**			0.131	0.876 (0.739 ~ 1.040)	0.921	1.010 (0.835 ~ 1.221)
Very heavy	1753	109 (6.2)				
Heavy	2885	157 (5.4)				
Average	624	30 (4.8)				
Small	64	4 (4.9)				
Very small	18	0 (0.0)				
**Workload compared with income**			0.096	0.889 (0.774 ~ 1.021)	0.855	1.018 (0.840 ~ 1.234)
Workload far greater than income	652	50 (7.7)				
Workload greater than income	1825	102 (5.6)				
Balanced	2447	123 (5.0)				
Workload less than income	335	19 (5.6)				
Workload far less than income	85	6 (7.1)				
**The essence of doctors’ work**						
Make a profit	129	8 (6.2)		1		1
Service sector	2048	89 (4.3)	0.324	0.687 (0.326 ~ 1.449)	0.393	0.714 (0.329 ~ 1.547)
Technical work	746	49 (6.6)	0.876	1.063 (0.491 ~ 2.301)	0.717	1.159 (0.521 ~ 2.583)
Help others	2421	154 (6.4)	0.942	1.027 (0.493 ~ 2.140)	0.924	1.038 (0.484 ~ 2.227)
**Doctor-patient relationship**			0.010	1.215 (1.048 ~ 1.409)	0.028	1.187 (1.018 ~ 1.384)
Very tense	88	3 (3.4)				
Tense	1135	60 (5.3)				
Neutral	2574	131 (5.1)				
Harmonious	1434	92 (6.4)				
Very harmonious	113	14 (12.4)				

*CILCM = clear intention to learn clinical medicine

Students who will definitely apply for clinical medicine are defined as having a CILCM

Students were obviously unevenly distributed in their opinion on social status and career prospects. More than half (2999/5344, 56.1%) of the students were optimistic about the career prospects of doctors, and 65.8% (3517/5344) of the students thought doctors had high or very high social status. Among the students who were very optimistic about career prospects, 35.3% (106/1044) of them had a CILCM. Basic information was added as correction factors in the multivariate analysis again. Students’ cognition of doctor’s career prospects, social status, and doctor-patient relationship influenced their intention to learn medicine. Those who were optimistic about doctors’ career prospects (*P* < 0.001, OR = 1.664) and those who felt that doctors had a high social status (*P* = 0.013, OR = 1.254) were more likely to choose clinical medicine.

Not surprisingly, over 80 % (4638/5344, 86.8%) of the students thought that the workload of doctors was heavy or very heavy. In terms of doctors’ income, 2477 (46.4%) students felt that doctors’ workload was greater than their income, and this was 4.89 times higher than that for students with opposite opinions (that doctors’ workload is less or far less than income). The largest number of students thought that helping others was the essence of doctors’ work, followed by the service sector, technical work, and making a profit. No correlation was found in either univariate or multivariate regression between students’ opinion on the essence of doctors’ work, workload, and workload compared with income and CILCM.

A total of 1547 (28.9%) of students thought that the doctor-patient relationship was harmonious or very harmonious. The univariate analysis revealed that students’ attitude towards the doctor-patient relationship was significantly associated with their CILCM (*P* = 0.010, OR = 1.215), which is similar to the association identified in the multivariate analysis (*P* = 0.028, OR = 1.187).

## Discussion

Senior high school students are the potential and basic talent reserve for the health sector. It is crucial to start with the root of this problem and study these students. Our findings showed that only 5.6% (300/5344) of the respondents had a CILCM, which was markedly lower than the values reported in previous domestic studies [16]. We have shown that the characters of students with a CILCM: female, senior ones (the third year),whose parents are medical workers and support their medical learning. Students’ CILCM was influenced by a variety of factors, some of which are slow to change (medical interest, ect) and some of which are modifiable, for example, the degree of understanding of the duration of medical schooling.

Consistent with previous findings, gender was found to have a significant influence on students’ choice of medicine [19–21]. In this research, the situation was the same. The results showed that more females preferred to be a doctor than their male peers. Due to the prevalence of gender-science stereotypes in all cultural views, most females have low interest and performance in science, technology, etc. [22] To be a doctor is more congruent with traditional gender-role stereotypes and meets the needs of females’ high prospect of financial security. In addition, females typically are more empathetic and have superior communication skills, which are important for the medical profession [23]. The potential impact of this trend on the growing number of women in medicine-related fields deserves further attention, which will be helpful to anticipate the future medical workforce. Compared with students in other grades, more senior students (the third-year students) have a CILCM. The findings agree with other relevant findings: younger students are less motivated for medicine than older students. The probable cause could be that age and maturity facilitate definitive career choice [20].

In this study, the family income directly reflects the household economic situation in monetary scale and parents’ education level directly reflect family cultural background. Nevertheless, there were few differences in students’ medical intention on family income and parents’ education level. A possible explanation is that China has launched curricular strategy for rural coverage through new policies in both the education and health sectors, which reduces the cost of study and provides more opportunities for students from poor backgrounds [4]. Usually, a medical career is considered an elite profession and appears to be a social and economic mobility path for families in most families [24]. In previous studies, family expectations were considered an important motivation for choosing medicine, particularly amongst those from non-Western backgrounds [25–27]. This also agreed with our findings: the greater the support from family members, the more likely students were to learn clinical medicine. Students with medical professionals in the family might realise the importance of the medical profession, thus piquing their interest in medicine [25]. Similarly, those students more familiar with medical academic rules, communication, and herit the medical practice from one of their parents [28]. Such results echo what we found: students belonging to a medical family were more inclined to choose clinical study. It is reasonable to explain the strong influence of family backgrounds especially within the Chinese cultural context. However, there are findings suggesting that students with strong parental expectations appeared to be more ambivalent than others about their choice to study medicine and were questioning whether the personal costs of a career in medicine were too high [29]. Which type of family background will promote the career development of medical students and the development of health workforce deserves further attention. More research is needed on this issue.

In line with previous studies, [30, 31] we found that interest in medicine motivated the students to choose medicine. Motivational theories suggest that students who have higher interest in learning medicine may have higher academic achievement and a higher level of professional identity [30, 32]. Career theories argue that career satisfaction and continuance are determined by one’s personal vocational interests and values [33]. To cultivate productive, engaged and satisfied doctors, the first step is that guide students to choose clinical medicine according to their interests. Our findings showed that the 53.8% (2875/5344) of participants were lacking the information about the long duration of schooling for clinical medicine, which seriously hindered their clinical medicine pursuit. Similarly, a total of 37.6% (2008/5344) of students reported that an anatomy course adversely affected their choice of clinical medicine. Some studies have found that the most prominent factor that influenced students’ autonomous motivation and strength of motivation was experiencing or gaining knowledge about the medical profession [25]. Thus, exposure to current clinical medicine education and training programs is crucial.

Within our study we were able to show that senior high school students hold different views on the healthcare occupation, which affected their medical choice. “Career prospects” and “Social status” were considered as the most important factors. A study in Finland from 1977 to 2006 suggested that most young doctors value career development and education [34]. We also found that students who were optimistic about the career prospects of doctors were more inclined to pursue medical careers. Optimistic career prospects represent not only good job security but also the development of professional ability and sense of achievement. The results corresponded well to previous studies: the main motivators to select medicine by medical students of upper-middle income countries were job security, while the main motivation of medical students in high-income countries were to develop specialized skills [35]. Our results showed that approximately three fourths of the respondents believed that doctors have high social status, and their intention to study clinical medicine was obviously more clear. Previous studies have proved the fact that the medical profession is preferred by the students due to its high social status [36, 37]. Social status is described by middle zone in the Maslow’s hierarchy of needs pyramid were identified, and has become the main reasons why students from low-middle income countries and upper-middle income countries choose medicine [35]. All aspects of security is a powerful factor for choosing medical learning. These are the driving force that can be used to attract the students into medical profession, hence improving the workforce. Our results showed that 45.3% (2421/5344) of participants thought that the essence of doctors’ work is helping others, and 38.3% (2048/5344) thought it is serving society. Helping others and serving society can be classified as altruism and it is the motivation for choosing a medical career [38, 39]. Previous studies have reported that medical students with the altruistic motivation are more likely to consider future involvement in medical work and show less jog burnout [38, 40]. Engendering humanistic consciousness of senior high school students might lead more talents to enroll into medical career and serve the humanity. Escalating tension in DPR have become a common phenomenon all over the world, especially in developing countries [41, 42]. Taking China as an example, nearly 60% of medical staff in China had experienced verbal abuse, and around one in seven had been physically assaulted according to a Chinese Medical Doctor Association report in 2015 [43]. This situation will bring great pressure to both doctors and medical students, resulting dismission of doctors and loss of medical students [42]. Similarly, the results showed that senior high school students’ negative attitude towards DPR was correlated with no intention to pursue medical careers and vice versa, similar to findings of previous studies [44, 45]. Unfortunately,we found that only a quarter of students possessed a positive attitude to DPR. Accordingly, improving DPR favours the doctors of today and also helps attract more senior high school students to become doctors of tomorrow [46].

Given that UHC has been identified as a priority for the global health agenda, the evidence from this study can be suggestive of furthering on in the UHC journey and taking the policy steps [47]. Firstly, the factors influence choice of clinical medicine are a combination of medical interests and knowledge about the medical profession. Therefore, teachers and parents should cultivate students’ professional interests and career selection, fully respect their decisions, and provide guidance on voluntarily applying for college. Medical colleges could stimulate students’ interest with more innovative strategies such as providing opportunities to volunteer in health service or participate in medical research [4, 48]. Health professional training institutions should enhance the promotion of clinical medicine-related information, establish and improve authoritative and objective information sources, and help senior high school students fully understand the training mode of medical education so that they can make rational choices based on sufficient information resources. Secondly, students’ cognition of health occupation and DPR play an important part in their CILCM. Measures to improve safety require significant health-care system changes to tackle the roots of tension DPR. Medical schools should help students cultivate their recognition of healthcare occupation and present DPR didactically through lectures. Social media should also play a more constructive role in harmonising the DPR by avoiding sensationalist coverage of violent events; setting high or professional standards of truth, accuracy, objectivity, and balance; inviting professional agencies and experts to elaborate the problem between the two parties; and carefully presenting images of both doctors and patients objectively and prudently [49, 50]. With the popularity of new media, it necessary to rationally use new media technology to strengthen scientific views and promote communication between the two parties to build doctor-patient trust. To some extent, by publicizing the role models of doctors and producing TV programmes (reality shows and documentaries), students’ cognition of health occupation can be improved and interests in medicine can be elicited, so as to choose clinical medicine [25].

This study has a few limitations. First, participants in this study were restricted to 10 senior high schools in 4 provinces. Although the provinces differ in economic and geographic location, the study sample may not be an accurate reflection of the whole population. Therefore, we expect that our study can be generalised to a larger student population. Second, clinical medicine does not represent the entire health field although it occupies a large proportion of medicine. Consequently, the findings from this study should be further investigated in all health-related subjects.

## Conclusions

The survey showed that only a minority of students have a CILCM. In addition to unchangeable interfering factors such as gender, age, and family background, students’ intention may grow through improving the current working condition of doctors, easing the doctor-patient relationship, and strengthening messaging about clinical medicine education for senior high school students. To some extent, this survey objectively and accurately reflects the dilemma of the talent reservoir in the medical and healthcare industries in China, providing a potential solution to this problem at an early stage. In addition, these findings provide new a perspective for understanding the development status of health services in developing countries.

## Supplementary Information

**Additional file 1.** Questionnaire

## Data Availability

The datasets used and/or analysed during the current study are available from the corresponding author on reasonable request.

## Abbreviations

CILCM: clear intention to learn clinical medicine
CEE: college entrance examination
DPR: doctor-patient relationship
UHC: Universal health coverage
