# Supplementary material for: An investigation of the intention and reasons of senior high school students in China to choose medical school
Source: BMC Med Educ. 2021 Apr 26;21:242. doi: 10.1186/s12909-021-02677-w (PMC8077942; doi:10.1186/s12909-021-02677-w)
Supplement: Supplementary file 1 — Additional file 1. Questionnaire [file 12909_2021_2677_MOESM1_ESM.pdf]

An investigation of the intention and reasons of senior high school students in China to choose enter into medical school

1.Gender □Male □Female
2.Grades □Senior one □Senior two □Senior three
3.What’s your academic performance ranking usually?
□Top 5%
□6%~ 30%
□31%~70%
□71%~100%
4.What is monthly income of your family? （RMB per person）
□<1000
□≥1000， <3000
□≥3000， <5000
□≥5000， <10000
□≥10000
5. Either or both of your parents are medical workers.
□Yes
□No
6.What’s your parents’ highest education background?
□Senior high school or below
□Undergraduate/junior college education
□Postgraduate or above
7. What is your family's attitude towards learning clinical medicine?
□Support
□Neutral
□Oppose
8.Will you apply for clinical medicine?
□Definitely I will
□Maybe
□Definitely I won’t
□I have no idea
9.Are you interested in medicine?
□Totally uninterested
□Uninterested
□Neutral
□Interested
□Very interested
10.You know the duration of clinical medicine schooling very well.
□Strongly agree
□Agree
□Neutral
□disagree
□Strongly disagree

11.Anatomy will influence your choice of major selection.
□Yes
□No
11.What’s your opinion about doctor’s career prospects?
□Very pessimistic
□Pessimistic
□Neutral
□Optimistic
□Very optimistic
12.What’s your opinion about doctor’s social status?
□Very high
□High
□Average
□Low
□Very low
13.Doctor is a job with great sense of achievement.
□Strongly agree
□Agree
□Neutral
□disagree
□Strongly disagree
14.What’s your opinion about doctor’s workload.
□Very heavy
□Heavy
□Average
□Small
□Very small
15.What’s your opinion about doctor’s workload compared with income?
□Workload far greater than income
□Workload greater than income
□Balanced
□Workload less than income
□Workload far less than income
16.What do you think of the essence of doctor’s work.
□Make a profit
□Service sector
□Technical work
□Help others
17.You are more impressive with the _____ of doctors shaped by the media.
□positive image
□negative image
18.Which kind of works that focuses on the medical sector do you prefer?
□Fictional film and television works
□Realistic documentary

19. What do you think of the current doctor-patient relationship in China?
□Very tense
□Tense
□Neutral
□Harmonious
□Very harmonious
20. How do you pick up information about the education of clinical medicine and the medical sector?
□I know nothing about education of clinical medicine and medical sector
□Traditional media(e.g. book)
□New media(e.g. network, app)
□School lecture
□Heard from people around

p.s. In our survey, this questionnaire is in Chinese. Although we try our best to keep the original meaning of the questionnaire, we’re worried that due to the different language habits between Chinese and English, the meaning of some items may be slightly deviated. So please contact us if you have any questions.
